# College Students’ Experience of a Food Safety Class and Their Responses to the MSG Issue

**DOI:** 10.3390/ijerph16162977

**Published:** 2019-08-19

**Authors:** Hyun Joung Jin, Dae Hee Han

**Affiliations:** 1 Department of Economics, College of Business & Economics, Chung-Ang University, Seoul 06974, Korea; 2 Department of Applied Health Science, School of Public Health, Indiana University, Bloomington, IN 47405, USA

**Keywords:** food additives, MSG, food safety class, risk perception, framing effect

## Abstract

This study examines whether students’ experience in a food safety class affected their responses to the monosodium glutamate (MSG) issue and to message framing. We differentiated students into two groups depending on their involvement in a food safety class. The data were collected through in-class surveys in South Korea. A structural equation model was used where the dependent variable was students’ intention to avoid MSG; the mediating variables were knowledge, trust, attitude, and risk perception; and the exogenous variable was class experience. A difference-in-differences scheme was used to analyze the interaction between class experience and message frame. Empirical results show that students who took the class had relatively more knowledge of MSG along with lower risk perceptions or fears of MSG and thus a reduced intention to avoid it. The class experience also affected their trust in overall food safety in the domestic market as well as in food-related institutions and groups. Students showed sensitivity to message framing, although the sensitivity did not statistically differ by students’ class experience status. Our results imply that cultivating students’ knowledge of food additives through a food safety class enables them to respond more reasonably toward food additives.

## 1. Introduction

Additives are used by the food industry to extend the shelf life of food, enhance consumer appeal, and ensure the safety and quality of food [1]. However, consumers often look at food additives with suspicion and try to avoid them [2]. Most consumers have little knowledge of food additives and perceive them as harmful to their health [3]. Consumer concerns over additives may affect food manufacturers who process food as well as policymakers who endeavor to communicate effectively on food safety issues.

A notable example of consumer mistrust and concern over food additives is the monosodium glutamate (MSG) issue in South Korea. This is the most longstanding and frequently discussed issue relating to food safety in the country, with the majority of consumers believing that the additive is harmful [4,5]. The MSG issue has continued for decades in the country because of continued assertions of its hazards by mass media and civil groups. 

The controversy over MSG first arose in Western countries. MSG is the salt form of glutamic acid, and it is used as a food additive because of its flavor-enhancing properties. Some people believe it causes a number of allergy-like symptoms, including but not limited to the following: MSG-induced asthma; migraine headaches; hives; swelling of the face, mouth, and tongue; nausea; and rhinitis. However, most of the evidence comes from anecdotal cases and dubious clinical studies. Decades of research has failed to demonstrate a relationship between MSG consumption and the development of serious reactions in most people. In 1987, the U.S. Food and Drug Administration (FDA) placed MSG in the category of “generally recognized as safe” (GRAS) [6], based on its extensive history of use in food before 1958 and on published scientific evidence. Other examples of GRAS substances include salt, sugar, spices, and vitamins [7]. The Joint Expert FAO/WHO Committee on Food Additives [8] classified MSG as an additive with an “ADI (acceptable daily intake) not specified,” indicating that no toxicological concerns arise from its use as a food additive.

The dose-dependent effects of food additives in humans are evaluated by the ADI and estimated daily intake (EDI). The maximum allowable dietary exposure to a food additive is related to ADI, which is based on effects of food additives observed in animal models; specifically, the no-observed-adverse-effect level (NOAEL) determined in animal studies from their lifetime exposure to the substance. EDI is an indication of cumulative exposure to a given substance and reflects the concentration of that substance in the food itself as well as the amount of consumption of the food. Both ADI and EDI are determined for each food additive. U.S. FDA approval of a food additive is contingent upon the stipulation that the sum of all EDIs of a food additive from all sources must not exceed the ADI. The ADI for MSG is 120 mg/kg/d [9].

Although the controversy over MSG seems to have abated in Western countries, it is still a matter of concern in South Korea. The Korea Food and Drug Administration [10] has accepted MSG as safe and has allowed its unlimited use in South Korea. According to Sah and Yeo [11] and Woo [12], Korean consumers with more limited knowledge of food additives tend to be more concerned because they do not understand scientific facts related to safety issues. 

This study investigated two research questions regarding the MSG issue in South Korea. The first issue covers knowledge cultivation effects on consumers’ responses to the additive issue. Previous studies have suggested that excessive concerns over controversial food safety issues can be mitigated by developing more reasonable risk perceptions based on enhanced consumer knowledge [13,14,15,16,17]. There may be several important mediators concerning the enhancement of consumer knowledge regarding food safety and hazards, such as media information, consumer education, or government publicity. In the present study, we focus on education opportunities. Positive effects of risk education on recipients’ knowledge of health risks and desirable risk behaviors have been suggested in the literature [18,19,20,21]. The second issue is related to the message frame of food safety news. News presented by mass media and from scientists’ opinions is important in food safety issues [22,23]. Separate from the cultivation of consumer knowledge, specific frames used in media coverage can play a significant role in responses by the public [24]. This suggests that consumer responses, and thus purchasing behaviors, may differ according to the framing of information conveyed by the media. Radley [25], Dorfman [26], Cohen et al. [27], and Jin and Han [13] showed that consumers’ cognition and responses are affected by message frames. In addition, food safety knowledge and message framing may interact with each other. Jin and Han [13] revealed that people with less knowledge about a food hazard will have higher levels of disturbance due to reports conveyed by mass media regarding a particular food safety issue. 

For empirical application of the research questions, we used data from a survey of college students. First, we investigated whether students’ experience in a food safety class affected their responses to the MSG issue. The content of the class included food hazards, scientific facts about food additives, and the role of food-related institutions, including the government. Students’ class experience could have resulted in a higher level of knowledge of MSG through enhanced understanding of scientific facts related to safety issues and/or increased personal interest in food additives, although the class did not specifically cover the MSG issue. Accordingly, we hypothesized that students who attended the food safety class would have relatively better knowledge of MSG than those who did not, making them less fearful of the additive. We used a structural equation model (SEM) to examine the relation process. 

Second, we analyzed students’ responses to message framing as well as the interaction between class experience and message framing. That is, we tested whether a message frame with different information constitutions changed students’ intention to avoid MSG and whether students who took the class were affected less by message framing than those who did not attend the class. Jin and Han [13] showed that message framing regarding food safety issues has an influence on college students’ responses. Participants of the study revealed divergent purchase intentions in response to different headlines and different amounts of information within articles. Those who had less knowledge had more variation in their purchase intentions in response to different message frames. Here, we implemented different message frames regarding the MSG issue. We provided a short article with both positive and negative information regarding MSG to half of the survey respondents and another article with only positive information to the other half, producing a 2 × 2 factorial design, with class experience. The hypotheses tested were that different message frames cause different responses by the students and that the group who had taken the class would be less affected by the message framing.

The remainder of this paper is organized as follows: Section 2 presents the analytical methods and data; Section 3 presents the estimation results and discussions; and Section 4 concludes the paper.

## 2. Methods and Materials

### 2.1. Specification of Structural Equation Model

**Figure 1:**
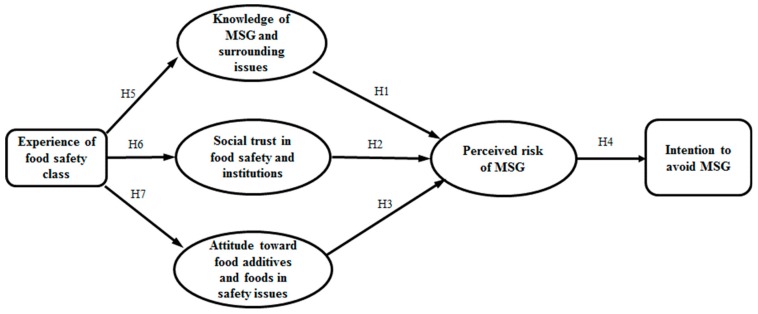
Specification of structural equation model.

For the first research question, this study used a reasoning process by which consumers’ responses to safety issues could be explained by knowledge, trust, attitude, and risk perception. The process was adopted from previous studies, such as Chen and Li [28], Lobb et al. [29], and Costa-Font and Gil [30], that extended Fishbein’s [31] multi-attribute attitude model. The dependent variable was students’ intention to avoid MSG (int-avoid), and the mediating variables were their social trust in the overall food safety of the domestic market and food-related institutions and groups (social trust), attitude toward food additives and foods in safety controversies (attitude), knowledge of MSG and surrounding issues (knowledge), and level of perceived risk of MSG (risk perception). The exogenous variable was the experience in the food safety class (class experience). The process was estimated using SEM as presented in Figure 1.

#### 2.1.1. Consumer Knowledge

Frewer et al. [32] maintained that consumers’ knowledge of food and safety plays an important role in their risk perception and that knowledge of food hazards can reduce sensitivity to food safety issues. This is consistent with the findings of previous studies on genetically modified (GM) food. Gaskell et al. [33] and Scholderer and Balderjahn [34] argued that limited knowledge of GM foods increased consumers’ perceptions of risk and reduced their acceptance of GM food. For the effect of prior knowledge on risk perception, therefore, we propose the following hypothesis.

**Hypothesis 1** **(H1).** *More knowledge of MSG and surrounding issues will reduce students’ perceptions of the risk of MSG.*

We measured students’ knowledge using subjective knowledge. Flynn and Goldsmith [35] suggested that subjective knowledge can convince consumers of the benefits of a product and that its effect is larger than that of objective knowledge. With regard to food choice, House et al. [36] showed that consumers’ subjective knowledge is positively associated with their willingness to accept GM food, but their objective knowledge is not. 

#### 2.1.2. Social Trust

According to Siegrist and Cvetkovich [37], when consumers face complexities regarding safety issues, their social trust becomes important because they cannot ensure the accuracy and reliability of information on hazards and risks. According to Siegrist [38], persons with higher social trust in institutions involved with gene technology have lower risk perceptions and foresee greater benefits of gene technology than those with lower social trust. Costa-Font and Gil [30] identified a similar tendency in three different European countries in that lower risk perceptions of GM food were accompanied by higher social trust.

Regarding the MSG debate, Korean students may not effectively assess the actual risks of MSG and therefore may depend on information from the government, the mass media, food manufacturers, and/or civil groups. Therefore, social trust should be an important factor in perceiving the risks of MSG. Thus, we propose the following hypothesis.

**Hypothesis 2** **(H2).** *Higher social trust in overall food safety in the domestic market and food-related institutions and groups will reduce students’ perceptions of risk regarding MSG.*

#### 2.1.3. Attitude

Attitude is one of the most crucial factors for predicting consumer choices across products and services [39]. Defined as a relatively permanent and stable evaluative summary of an item, it constitutes an important psychological construct because it has been found to influence and predict many behaviors [40]. Several studies have investigated the relationship between consumer attitudes and risk perceptions. Dickson-Spillmann et al. [41] showed that consumers with less negative attitudes to chemicals revealed lower risk perceptions of food additives and contaminants. Similarly, Costa-Font and Gil [30] found that consumers in Spain, Italy, and Greece who showed positive attitudes to new technology were likely to have lower risk perceptions of GM food. Thus, we propose the following hypothesis.

**Hypothesis 3** **(H3).** *Students with negative attitudes toward food additives and foods in safety controversies will have higher risk perceptions of MSG.*

#### 2.1.4. Risk Perception 

The risk perception of a food hazard is a psychological interpretation of the hazard. Yeung and Morris [42] identified an inverse relationship between British consumers’ perceptions of a microbiological risk in chicken and their willingness to purchase it. Similarly, Costa-Font and Gil [30], Grunert et al. [43], and Honkanen and Verplanken [44] showed that consumers’ purchase intentions regarding GM food are negatively influenced by their risk perceptions of GM food. Therefore, we propose the following hypothesis:

**Hypothesis 4** **(H4).** *Students with higher risk perceptions of MSG will have a greater intention to avoid it.*

#### 2.1.5. Experience of Food Safety Class 

We extended the SEM model by including “experience in a food safety class.” We expected that the class experience would affect knowledge, social trust, and attitude, and that it would indirectly affect risk perception and int-avoid as a result. First, students who attended the class were expected to have more knowledge of MSG and surrounding issues than those who did not. Second, the experience of the class was expected to affect the students’ social trust in the overall safety of food in the domestic market and related institutions and groups. The sign of the relationship could not be predicted because it was not clear whether the class experience would increase their trust. Third, we expected that students who attended the class would have less negative attitudes toward food additives and foods involved in safety controversies than those with no class experience, as studying scientific facts of hazards would develop the students’ scientific risk understanding, as suggested by Ronan and Johnston [19]. Accordingly, we propose the following three hypotheses.

**Hypothesis 5** **(H5).** *The experience of the food safety class will have a positive effect on students’ knowledge level regarding MSG.*

**Hypothesis 6** **(H6).** *The experience of the food safety class will have a positive effect on students’ social trust in food safety and related institutions.*

**Hypothesis 7** **(H7).** *The experience of the food safety class will have a positive effect on students’ attitudes toward food additives and foods in safety controversies.*

### 2.2. Test for Interaction with Message Frame

#### 2.2.1. Message Frames

Kahneman and Tversky [45] demonstrated how the framing effect works by choosing, omitting, and stressing a certain characteristics of a social phenomenon. The message frame in this study pertained to a case of omitting. In the survey process, respondents were first asked about knowledge, social trust, attitude, and risk perception. Next, a short news article about MSG was provided before asking about their intention to avoid MSG. Respondents were shown one of two different types of articles: either an article with both positive and negative information regarding MSG (frame B) or one with only positive information regarding MSG (frame A). That is, sentences conveying negative information were omitted from frame A but included in frame B as detailed in Table 1.

#### 2.2.2. Estimation Model

Analysis for the interaction between the class experience and message framing can be completed through a difference-in-differences (DID) frame [46,47]. We compare students’ responses to the message frames according to different groups classified by their class involvement. Students who took the class belong to the treatment group, and students who did not take the class belong to the control group. In principle, the control group shows what would have happened to the treatment group in the absence of any treatment. To explain in detail, let ${\bar{y}}_{cm}$ be the average of students’ int-avoid, with class experience status, *c*, and message frame status, *m*. The value of c would equal 1 if individual *i* took the class or 0 if he or she did not, and the value of *m* would equal 1 if individual *i* received frame B or 0 if he or she received frame A. The definition of DID provides the estimator of treatment effect, $\hat{\delta }$, as follows:

(1) $$\hat{\delta }=({\bar{y}}_{00}-{\bar{y}}_{01})-({\bar{y}}_{10}-{\bar{y}}_{11}).$$

**Figure 2:**
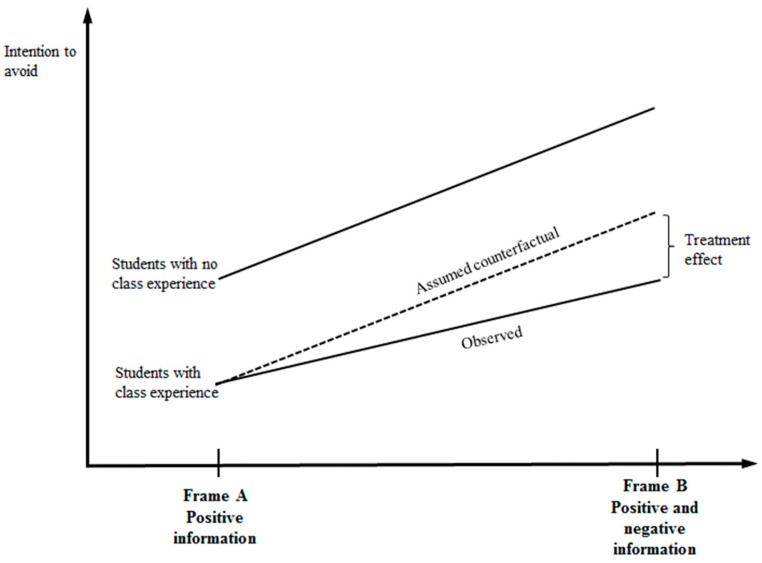
Estimation strategy for the treatment effect based on assumed counterfactual.

The estimation strategy for the treatment effect based on the assumed counterfactual is presented in Figure 2. It is expected that students’ int-avoid value will be higher when faced with frame B compared to when faced with frame A, irrespective of the class experience, since frame B has negative information as well as positive information. The DID assumes that the slope changes from frame A to frame B will be the same for both groups unless there was a treatment effect, which is the parallel assumption. If the slope of the treatment group differs significantly from what is expected by the parallel assumption, it is said to find a differential effect between the “treatment group” and the “control group.” In the figure, the treatment effect is the difference between the observed value of the int-avoid of the treatment group and the value derived by the assumed counterfactual, had there been no class experience. The value of the difference can be either positive or negative, although the figure illustrates an example of smaller observed value than the assumed counterfactual value. 

The $\hat{\delta }$ in Equation (1) can be estimated in a linear equation form as follows:

(2) $$y_{cmi}=\beta_{0}+\beta_{1}c_{i}+\beta_{2}m_{i}+\delta (c_{i}\times m_{i})+X_{cmi}\theta +e_{cmi}$$

where $c_{i}$ and $m_{i}$ are dichotomous variables for class experience and message frame, respectively. X is the vector of individual factors. The parameter regarding the interaction between $c_{i}$ and $m_{i}$, $\hat{\delta }$, which measures the int-avoid level of students who took the class and faced frame B, is of particular interest. Instead of the DID form in Equation (2), one can use a control variable method or a comparison of respectively estimated $\beta_{2}$ for both the treatment group and the control group. The dependent variable was measured using a five-point scale. Therefore, we estimated Equation (2) using an ordered logistic regression instead of ordinary least squares (OLS). For individual factors, we included gender, age, university year, disease status of household members, and proportion of discretionary income spent on food. For the disease status of household members, if at least one household member has an illness, atopic dermatitis, high blood pressure, diabetes, or cancer, we classified it as “yes,” otherwise, it was classified it as “no.” 

### 2.3. Participants and Measures

The data for this study were obtained from a survey conducted in a university in Seoul, South Korea from 20 May to 1 June, 2019. All students participated voluntarily after providing consent. The students were asked to submit the questionnaires anonymously. The Korean Statistics Act and the purpose of the survey were explained to the students in the introductory part of the questionnaire. Out of the total 300 responses, 17 were found to have incomplete answers to the survey questions. Therefore, the final number of observations was 283. Table 2 summarizes the participants’ demographic characteristics.

Table 3 details the measures for the constructs. The objects of trust were divided into five categories: overall food safety in the domestic market, government and media reports, government policies and practices, food manufacturers, and civil groups. The measure for knowledge of MSG was adopted from Flynn and Goldsmith [35]. All five questions were measured on a five-point Likert scale ranging from “strongly disagree” to “strongly agree”. 

## 3. Results and Discussion

### 3.1. Results of SEM Estimation

We conducted a confirmatory factor analysis (CFA) before estimating the SEM model to assess the construct validity and reliability of the measurement model. Further, we checked the construct validity by examining the convergent validity and discriminant validity. The results indicated statistical significance of the t-values associated with standardized loadings (*p* < 0.01), suggesting a sufficient degree of convergent validity. The results also confirmed that no squared correlation exceeded the extracted average variance, indicating that the criterion for discriminant validity was met. In addition, all the Cronbach’s alpha and composite reliability values were above 0.7, indicating acceptable reliability [48]. Overall, the reliability and validity analysis results indicated satisfactory measurement qualities. The model fit statistics showed that χ^2^/df was 3.35 (which is close to 3), the normed fit index (NFI) was 0.795, the comparative fit index (CFI) was 0.837, the Akaike information criteria (AIC) was 64.83, and the root mean squared error of approximation (RMSEA) was 0.091 (which is close to zero). The results suggested a reasonable goodness-of-fit for the model [49]. The estimation results are shown in Table 4. 

The SEM results showed that students with more knowledge of MSG demonstrated lower levels of risk perception (supporting H1). However, students’ social trust did not reveal any influence on risk perception (not supporting H2). Attitude negatively affected risk perception (supporting H3). Specifically, it showed that if students had more negative attitudes toward food additives and foods in safety controversies, they perceived MSG as riskier. Int-avoid was influenced by risk perception (supporting H4), suggesting that students are more likely to avoid MSG when their risk perception of MSG is relatively higher. We further found that class experience increased students’ objective knowledge of MSG (supporting H5). Class experience also had a positive impact on participants’ social trust in food safety and related institutions and groups (supporting H6). However, class experience did not show a statistically significant influence on the students’ attitudes toward food additives and foods in safety controversies (not supporting H7).

We further derived the indirect effect of class experience on risk perception and int-avoid. Using the bootstrap method, we derived the standard error of the indirect effect and therefore, the statistical significance of each effect. The findings in Table 5 show that students with the class experience showed less risk perception regarding MSG and less intention to avoid MSG.

### 3.2. Results of Ordered Logit Estimation

The results of the ordered logistic regression are presented in Table 6. Since there was a relatively large number of explanatory variables, the multicollinearity among the variables was checked using the variance inflation factor (VIF). All estimated VIFs were less than 2.0, meaning that multicollinearity was not a significant concern in the estimation.

As presented in the table, there are three different models: (1) a model with only the variables of class experience, message frame, and class experience × message frame, (2) a model in which demographic variables were added to Model 1, and (3) a model in which social trust, knowledge, attitude, and perceived risk were added to Model 2. Class experience and message frame were statistically significant throughout all models. Class experience worked to decrease the int-avoid value. Message frame worked to increase the int-avoid rate, meaning that when students were provided with both positive and negative information regarding MSG, their intention to avoid MSG increased compared to the case in which only positive information was provided. This means that the message frame had an influence on the students’ responses as measured by purchase intention. 

The coefficient of class experience × message frame, $\hat{\delta }$, had a negative value, which means that the observed int-avoid value of the treatment group, when faced with frame B, is smaller than the value derived by the assumed counterfactual. That is, the responses to the message frame by students who took the class were weaker than those from students who did not take the class. However, the coefficient was not statistically significant at any conventional significance level throughout the models, which means that different responses to the message frame, according to different status of class experience, were not statistically supported. This may be because the college students were sensitive to the message framing itself, regardless of their class experience status, or because we provided a strong form of message framing: positive information versus positive and negative information together. If we used other message frames such as different information details or different nuance in headlines, we might have obtained statistically significant support for different responses to message frames based on the class experience.

### 3.3. Discussion

Information and education would be important mediators in enhancing consumer knowledge regarding MSG. An educational effect on the MSG issue has not been found in the literature. However, we did find studies analyzing the role of MSG information in shaping consumer responses, and the findings of those studies are inconclusive. Wang and Adhikari [50] performed a cross-sectional survey examining U.S. consumers’ perceptions of MSG and found that consumers who have information about MSG are more likely to report higher risk perception of MSG than their counterparts. Conversely, Greenacre et al. [51], using an in-depth online panel survey through Amazon’s Mechanical Turk, found that factual information in the shape of a rational appeal played a crucial role in increasing the likelihood of consuming MSG, suggesting the importance of providing accurate health information based on the true nature of MSG. A key difference between these two studies is the nature of the information respondents have or were provided; that is, subjective/emotional versus objective/rational. This implies that both the way information is conveyed as well as what information is conveyed are important, which is related to our second research question.

Our second result indicated the sensitivity of students’ responses to message framing, although sensitivity did not differ statistically by whether students had the class experience. This confirms that the delivering frame of food safety news is important, suggesting that specific frames used in media coverage can play a significant role in responses by the public. This implies that consumer responses, and thus purchasing behaviors, may differ according to how and/or what information is conveyed by the media. The findings have implications to which the government, mass media, and food makers need to pay attention when providing news or information regarding food hazard and safety. Additionally, the food industry and policymakers need to communicate with consumers with objective and correct information, and they need to educate consumers on a factual basis regarding MSG.

The results of our first research question are aligned with those of Greenacre et al. [51], given that opportunity to participate in a food safety class provides recipients with more knowledge of food hazards and safety and thus make their perceptions of controversial food safety issues more reasonable. Greenacre et al. [51] imply that food manufacturers need to place correct information about the true nature of MSG in their advertising and on their food products rather than allowing incorrect beliefs to pervade. This finding, together with our results, imply that in order to reduce unnecessary consumer concern, consumers’ knowledge of food additives and scientific facts need to increase through education on a factual basis. Shim et al. [52] reported that 76.8% of South Korean respondents felt information on food additives is insufficient. They attributed this lack of information to difficulties in understanding the subject of food additives as well as insufficient education and public relations campaigns. Therefore, our results suggest recommendations for regulatory agencies and food-related industries—they need to develop an educational curriculum related to food additives and scientific facts, in addition to transmitting information regarding additives to the public. If governments, schools, or other relevant institutions or groups could provide the public with opportunities to participate in food safety education, recipients would have a better understanding of such issues, and it would result in more realistic risk perceptions and rational responses to food safety issues. 

## 4. Conclusions

We analyzed whether students’ experience in a food safety class would result in more realistic responses to the controversial MSG issue. Our findings suggested that although students with class experience did not specifically discuss the MSG issue, they had relatively more knowledge of MSG and a lower risk perception or fear of MSG, resulting in a reduced intention to avoid MSG. Their class experience also increased their trust in overall food safety in the domestic market as well as in food-related institutions and groups. Students showed sensitive responses to message framing, but the sensitivity did not statistically differ by whether students had the class experience. This may be because college students were sensitive to the message frames regardless of their class experience status or because we provided a strong form of message frame. 

This study has several limitations, which lead us to provide certain suggestions for future studies. First, because the survey data were restricted to college students, discretion is required before generalizing the findings to other groups. Second, the findings we derived relate to only one country. Future analyses should examine various countries to arrive at a strong conclusion. Several important control variables, such as personal hazard experience, optimistic bias, and other demographic characteristics, may interact and raise consumers’ knowledge of food additives and desirable risk behaviors, in addition to education. Therefore, future studies should focus on these other factors as well. 

## Figures and Tables

**Table 1:** Difference in messages between frame A and frame B.

Frame A	Frame B
Is MSG truly detrimental for our health? The safety of the artificial flavor enhancer MSG has been a controversial issue for a long time. In 1987, according to the Joint FAO/WHO Codex Alimentarius Commission, the safety of MSG at normally consumed levels for the general population was proved, but like sodium and sugar, it should be used in as small an amount as possible in foods.	Is MSG truly detrimental for our health? The safety of the artificial flavor enhancer MSG has been a controversial issue for a long time. In 1987, according to the Joint FAO/WHO Codex Alimentarius Commission, the safety of MSG at normally consumed levels for the general population was proved, but like sodium and sugar, it should be used in as small an amount as possible in foods. The Federation of American Societies for Experimental Biology (FASEB) admitted that certain people may develop short-term reactions (chest pain, headache, nausea).

**Table 2:** Demographic characteristics of participants.

Characteristics		No.	%	Characteristics		No.	%
Gender	Male	121	42.8	Discretionary income per month	0–200	38	13.5
	Female	162	57.2		201–300	105	37.1
Age	Under 23	96	33.9		301–400	70	24.7
	23–25	145	51.2		Over 400	70	24.7
	26 and older	42	14.9	Proportion of discretionary income spent on food	Less than 20%	10	3.5
Disease status of household member	Yes	87	30.7		20–39%	67	23.7
	No	196	69.3		40–59%	119	42.1
Experience in class	Yes	146	51.6		60–79%	72	25.4
	No	137	48.4		More than 80%	15	5.3
Monthly household income	Under 1000	4	1.4	Year	Sophomore	29	10.2
	1000–2999	46	16.3		Junior	155	54.8
	3000–4999	93	32.9		Senior	99	35.0
	5000–6999	74	26.1	Message frame	Type A	141	49.8
	7000–8999	36	12.7		Type B	142	50.2
	Over 9000	30	10.6	Total	283		

Notes: The monetary unit of income and discretionary income is thousands of Koreans won. The exchange rate at the time of the survey was US$1 equal to 1188 Korean won (24 May, 2019).

**Table 3:** List of indicators used to measure each construct.

Construct	Indicator
Knowledge regarding MSG issue	I know more about MSG than most other people.
	Among my neighbors and friends, I am a quasi “expert” on the MSG issue.
	I can explain MSG and its risks pretty well using scientific facts.
Trust in overall food safety and food-related institutions and groups	I trust in the overall food safety of the domestic market.
	I trust in the government announcements and media reports regarding food safety issues.
	I believe that the government policies regarding food safety are relevant.
	I think that food manufacturers follow food safety procedures.
	I believe in the opinions of civil groups regarding food safety issues.
Attitude toward food additives and foods in safety controversies	I look at food additives with suspicion.
	I suspect the safety of foods in safety controversies regardless of their authenticity.
	I try not to consume food additives and foods in safety controversies.
Perceived risk of MSG	I think MSG has a negative effect on human health.
	I may feel anxious if I eat food containing MSG.
	If I purchased food containing MSG, my family members would dislike it because it is related to a food safety problem.
	MSG became a social issue because of its risks.
Intention to avoid MSG	If possible, I will make every effort to avoid MSG.

**Table 4:** Estimation results of structural equation model.

Path	Coefficient	Standard Error	Critical Ratio (*t*-Value)
*H1:* Knowledge → Risk perception	−0.170 ***	0.065	−2.62
*H2:* Social trust → Risk perception	−0.068	0.062	−1.09
*H3:* Attitude → Risk perception	0.509 ***	0.091	5.57
*H4:* Risk perception → Int-avoid	0.074 ***	0.018	4.17
*H5:* Class experience → Knowledge	2.135 ***	0.302	7.07
*H6:* Class experience → Social trust	0.715 **	0.339	2.11
*H7:* Class experience → Attitude	0.195	0.232	0.84

Note: Asterisks ** and *** indicate statistical significance at the 5 percent and 1 percent levels, respectively.

**Table 5:** Decomposition of effect of class experience on other constructs.

Path	Total Effect	Direct Effect	Indirect Effect
Class experience → Knowledge	2.135	2.135 ***	-
Class experience → Social trust	0.715	0.715 **	-
Class experience → Attitude	0.195	0.195	-
Class experience → Risk perception	−0.311	-	−0.311 ***
Class experience → Int-avoid	−0.023	-	−0.023 ***

Note: Asterisks ** and *** indicate statistical significance at the 5 percent and 1 percent levels, respectively.

**Table 6:** Estimation results of ordered logistic regression.

Variable	(1)	(2)	(3)
Threshold 1	−2.453 ***	−2.134	−1.676
Threshold 2	−0.556 **	−0.211	0.419
Threshold 3	1.022 ***	1.390	2.164
Threshold 4	3.640 ***	4.012 *	4.892 *
Class experience	−0.578 * (0.304)	−0.784 ** (0.323)	−0.633 * (0.341)
Message frame	0.706 ** (0.316)	0.627 ** (0.318)	0.808 ** (0.326)
Class experience × Message frame	−0.182 (0.434)	−0.060 (0.445)	−0.126 (0.453)
Gender		0.262 (0.287)	0.126 (0.298)
Disease status of household member		−0.311 (0.242)	−0.276 (0.249)
Age		0.002 (0.096)	−0.008 (0.099)
University year		0.260 (0.218)	0.155 (0.223)
Proportion of discretionary income spent on food		−0.125 (0.129)	−0.175 (0.134)
Social trust			−0.123 *** (0.041)
Knowledge			−0.051 (0.046)
Attitude			0.179 *** (0.064)
Perceived risk			0.121 *** (0.040)
Likelihood ratio χ^2^ (*p*-value)	17.78 (0.000)	22.76 (0.000)	59.16 (0.000)
Log likelihood	−31.93	−327.53	−360.94
Akaike information criterion (AIC)	77.852	679.065	753.887

Notes: Asterisks *, **, and *** indicate statistical significance at the 10%, 5%, and 1% levels, respectively. The values in parentheses denote standard errors. We set class experience to be 1 if students took the class or 0 if they did not, and we set the message frame to be 1 if students received frame A or 0 if they received frame B. The base group’s gender and disease status of household members are “male” and “yes,” respectively.
